# Asymptomatic ventriculomegaly in myelomeningocele: a matched cohort study of neuropsychological outcomes across institutions with varying shunt rates

**DOI:** 10.1007/s00381-025-06847-9

**Published:** 2025-05-30

**Authors:** Robin M. Bowman, Rya Muller, Jonathan Scoville, Adrien M. Winning, Allison D. Payne, Alexa Fagan, Caitlin Murray, Jaclyn L. Papadakis, Theresa Meyer, Jack M. Fletcher, Grayson N. Holmbeck

**Affiliations:** 1 Division of Pediatric Neurosurgery, Department of Surgery, Ann & Robert H. Lurie Children’s Hospital of Chicago, Chicago, USA; 2 Department of Neurological Surgery, Feinberg School of Medicine, Northwestern University, Chicago, USA; 3 Abigail Wexner Research Institute at Nationwide Children’s Hospital, Columbus, USA; 4 Department of Pediatrics, The Ohio State University, Columbus, USA; 5 Department of Psychology, Loyola University Chicago, Chicago, USA; 6 Katy Psychological Services, PLLC, Houston, USA; 7 Center for Child Health, Seattle Children’s Research Institute, Behavior & Development, Seattle, USA; 8 Department of Anesthesiology & Pain Medicine, University of Washington, Seattle, USA; 9 The Pritzker Department of Psychiatry and Behavioral Health, Ann & Robert H. Lurie Children’s Hospital of Chicago, Chicago, USA; 10 Department of Psychiatry and Behavioral Sciences, Northwestern University Feinberg School of Medicine, Chicago, USA; 11 Department of Psychology, University of Houston, Houston, USA

**Keywords:** Myelomeningocele, Ventriculomegaly, Neurocognitive, VP shunt, Hydrocephalus

## Abstract

**Purpose:**

Hydrocephalus management in myelomeningocele (MMC) patients remains controversial. While most institutions recommend surgical intervention for enlarged ventricles, recent studies suggest asymptomatic ventriculomegaly may not require treatment. This study compared cognitive outcomes in MMC patients from different institutions with different hydrocephalus intervention rates.

**Methods:**

Participants with MMC were recruited from two sites: Site 1 (Chicago area; *N* = 41; selective shunting protocol) and Site 2 (Houston and Toronto; *N* = 342; historical approach to hydrocephalus management). The 41 Site 1 patients were matched to 41 participants from Site 2 based on age, gender, lesion level, and race. Neuropsychological testing assessed various cognitive domains. Independent samples *t*-tests compared outcomes between sites.

**Results:**

Site 1 had significantly lower shunt rates (55% shunt rate at Site 1; 83% shunt rate at Site 2). There were no significant differences in demographics or lesion levels between sites. Site 1 participants demonstrated significantly higher scores on several cognitive measures compared to Site 2, including the Purdue Pegboard (fine motor dexterity; *p* = 0.042), Stanford-Binet Quantitative Reasoning (quantitative reasoning to solve mathematical problems; *p* = 0.030), VMI (visuomotor integration; *p* = 0.034), WJ Letter-Word Identification (single-word reading; *p* = 0.026), and WJ Calculation subtests (math calculation problem-solving; *p* = 0.012).

**Conclusion:**

Neuropsychological outcomes were either similar across cohorts from institutions with different shunt rates or favored the clinic with the lower shunting rate. These findings suggest asymptomatic ventriculomegaly may not be associated with worse functional outcomes, potentially informing guidelines for hydrocephalus intervention in the MMC population.

## Introduction

There are approximately 1500 pregnancies affected by neural tube defects (NTDs) each year in the United States of America (USA) [1]. Myelomeningocele (MMC), the most severe form of NTD, is frequently complicated by hydrocephalus, defined as the abnormal, progressive accumulation of cerebrospinal fluid (CSF) within the brain. The introduction of ventricular shunting in the 1950s marked a pivotal moment in the management of hydrocephalus, dramatically reducing associated morbidity and mortality [2].

Currently, most pediatric neurosurgeons advocate for CSF diversion via a ventriculoperitoneal (VP) shunt or, more recently, an endoscopic third ventriculostomy (ETV) for all MMC patients with ventricular enlargement or symptomatic hydrocephalus. However, there are no universally accepted management criteria for asymptomatic ventriculomegaly in the MMC population, with treatment rates ranging from 40 to 91% of patients [3–10].

Shunt complications are common and occur more frequently in patients with MMC than those with other conditions [11, 12]. Shunt failure occurs in 52–64% of cases, most commonly due to infection [12, 13]. Furthermore, up to 40% of patients experience slit ventricle syndrome within 6 years of shunt placement [13]. On average, most patients require 2–3 revisions during childhood, with 95% requiring at least one revision [14].

Recent advances, such as fetal MMC closure, have led to a decrease in shunt placement rates in this population [3, 15]. Given the morbidity and mortality associated with lifelong shunt dependence, recent research has focused on refining criteria for asymptomatic ventriculomegaly intervention [16]. Studies by Chakraborty et al. and Tulipan et al. have suggested that stable ventriculomegaly may be well tolerated in the MMC population [5, 16].

The association between larger ventricular size and worse cognitive outcomes remains inconsistent in the literature [17–21]. While some studies have demonstrated associations between larger ventricles and decreased IQ scores, others have shown minimal to no difference in cognitive outcomes for patients with shunts compared to those with ETV, where the ventricular size tends to remain large [17–19, 22]. A recent prospective study by the Hydrocephalus Clinical Research Network (HCRN) found no association between ventricular size and outcome on 23 out of 25 cognitive tests [23].

Given the lack of consensus over whether ventricular size impacts functional outcome, this study compares the cognitive outcomes between two cohorts of MMC patients managed using different protocols for hydrocephalus intervention, one resulting in a higher rate of shunt placement and the other a lower rate. This comparison aims to provide insight into the impact of a more aggressive versus conservative hydrocephalus management strategy on long-term neurocognitive function in this patient population.

## Methods

### Participants and procedure

Participants were recruited from two sites: Ann & Robert H. Lurie Children’s Hospital of Chicago (Site 1) and three collaborating clinics in Houston and Toronto (Site 2; see below). Site 1 followed a selective shunting protocol (~ 55% shunt rate), while Site 2 used the historical approach to hydrocephalus management (~ 80% shunt rate). All sites received institutional review board approval.

#### Site 1

Participants were recruited from 2016 to 2019 during clinic visits or via invitation letters. Inclusion criteria: (1) myelomeningocele diagnosis, (2) ages 2–17 years, (3) English or Spanish proficiency, (4) primary caregiver involvement, and (5) ability to complete assessments. Data were collected by trained research assistants during in-person, 1–3-h clinic visits. Participants completed performance-based neuropsychological assessments and parents/caregivers completed questionnaires. Of the 66 eligible patients, 41 participated in the study. Of the eligible participants, 25 were not enrolled due to declined participation (7), scheduling difficulties (16), or incomplete testing (2).

#### Site 2

Site 2 participants were recruited from three collaborating clinics: Shriner’s Hospital for Children-Houston, the Spina Bifida Clinic at Texas Children’s Hospital, and the Hospital for Sick Children in Toronto (*N* = 342). These clinics participated in a long-term study (1999–2010) of children and adults with spina bifida and other etiologies of early hydrocephalus. Data were combined across these sites due to similar patient management practices. All three clinics followed children who predominantly received initial surgery at TCH or Sick Kids, but the basis for recruitment was participation in one of the three follow-up clinics. These patients largely received historical treatment approaches for hydrocephalus in the MMC population. Those with other etiologies of hydrocephalus were excluded in this analysis (see Fletcher et al., 2004; 2005; Hampton et al., 2011; 2013 for details on the larger samples) [24–27].

#### Demographic information on sites

Table 1 reports demographic information for each site. Site 1 included 41 families, with an average age of 9.41 years (*SD* = 4.12); most participants were female (61.0%) and White (65.9%). From Site 2, 41 families were included as a matched comparison sample. On average, youth in this subsample were 9.44 years old (*SD* = 4.21; range = 3–16 years) and most were female (61.0%) and White (68.3%).

**Table 1:** Child demographic and condition-related characteristics by site

Characteristic	Site 1 (*N* = 41)	Site 2 (*N* = 41)
Sex: female	25 (61.0)	25 (61.0)
Age	9.41 (4.12)	9.44 (4.21)
Race/ethnicity		
White	27 (65.9)	28 (68.3)
African-American/Black	4 (9.8)	5 (12.2)
Hispanic/Latino	6 (14.6)	7 (17.1)
Asian	2 (4.9)	1 (2.4)
Unknown/not reported	2 (4.9)	0 (0)
Lesion level		
Thoracic	2 (4.9)	2 (4.9)
Lumbar	28 (68.3)	29 (70.7)
Sacral	9 (22.0)	9 (22.0)
Unknown/not reported	0 (0)	1 (2.4)
Shunt present	**21 (51.2)**	**34 (82.9)**
Number of shunt revisions	3.48 (5.89)	2.35 (5.35)

Information presented as frequency (%) for categorical data, whereas mean (*SD*) presented for continuous data. Significant group differences (*p* <.05) are bolded

#### Matching process

**Fig. 1:**
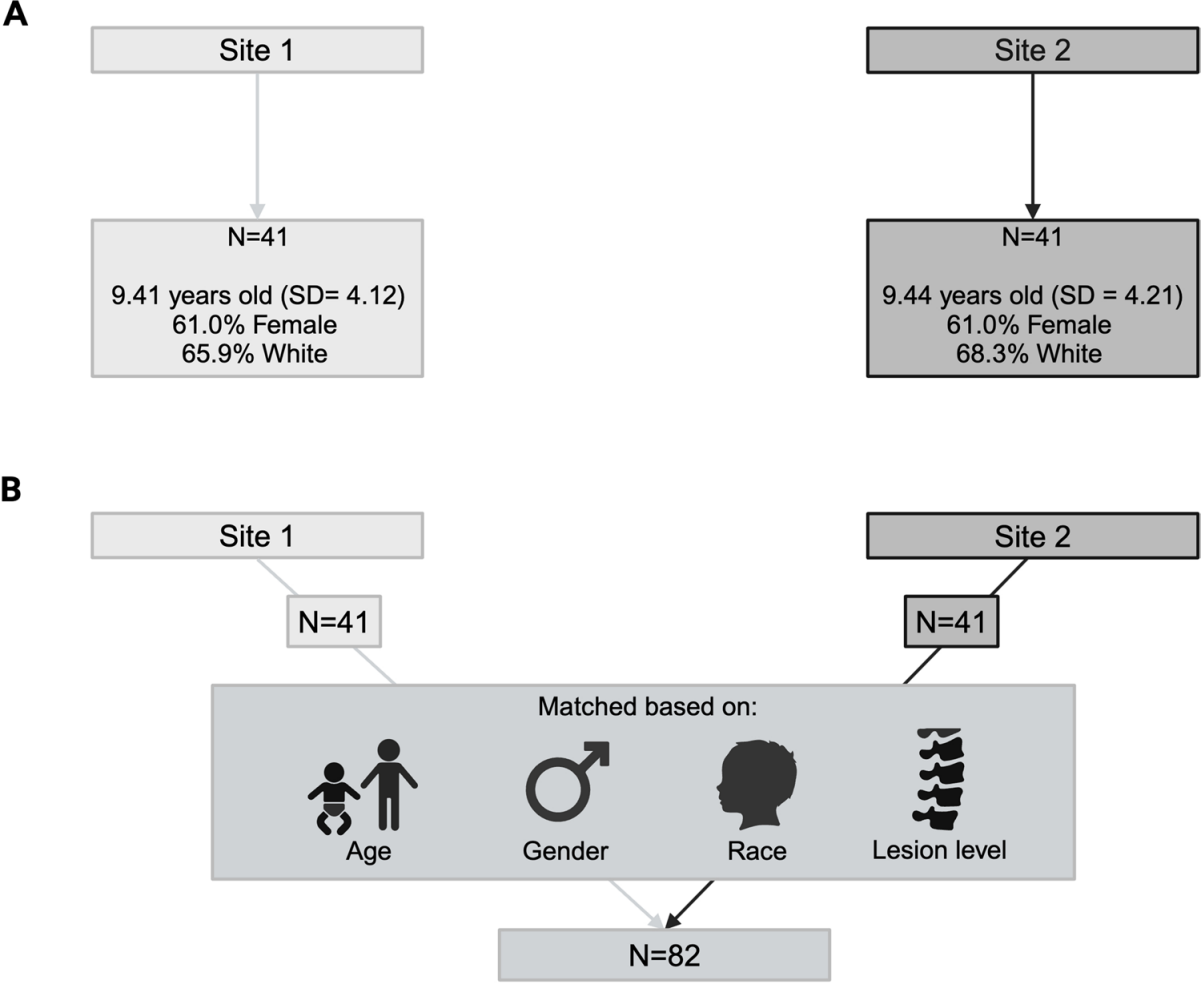
**A** Demographics across Site 1 and Site 2. **B** Matching process across the two sites. *N* = 41 patients from both Site 1 and Site 2 were matched based on age, gender, race, and lesion level. Created in BioRender. Bowman, R. (2024) https://BioRender.com/f86a915

Two matched samples were created (an overview of the matching process is shown in Fig. 1). Forty-one individuals from each site were matched based on age, gender, lesion level, and race. When exact matches were unavailable, criteria were relaxed in the following order: age (± 1 year), gender, race (White vs. non-White), and lesion level (sacral, lumbar, thoracic). To maintain group similarity, subsequent matches were adjusted to balance any deviations. If there was more than one possible match from Site 2 for a Site 1 participant, the matched participant from Site 2 was chosen randomly.

### Measures

**Fig. 2:**
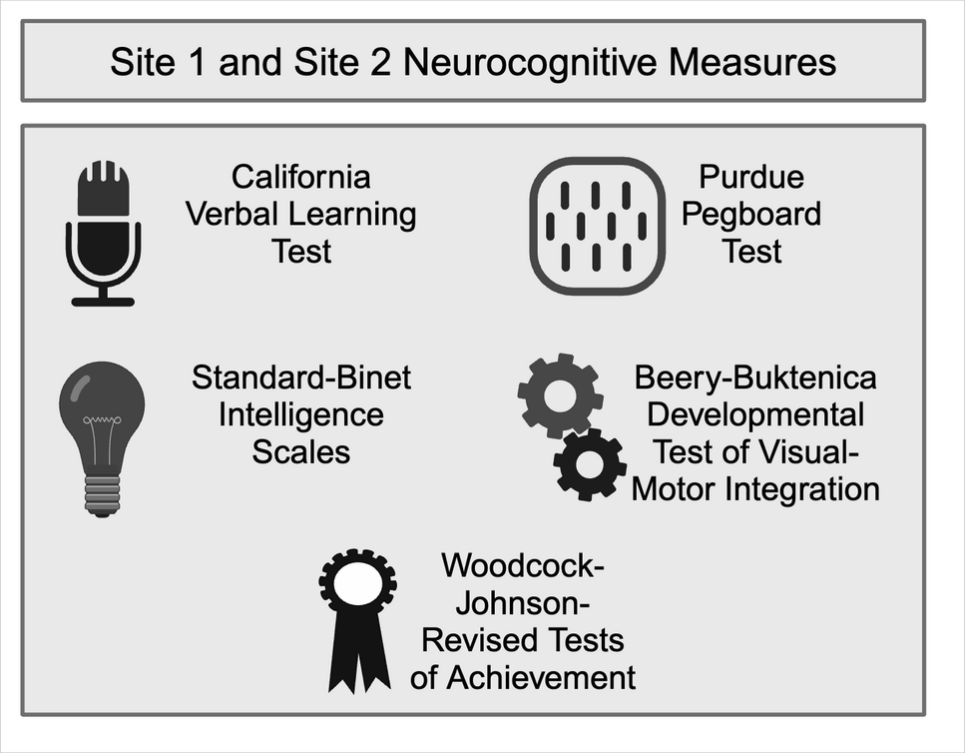
Neurocognitive measures conducted across Site 1 and Site 2. Created in BioRender. Bowman, R. (2024) https://BioRender.com/z26n144

Demographic information was collected via parental/caregiver questionnaires and medical chart reviews. Neurocognitive measures used at Site 1 and Site 2 are detailed in Fig. 2.

#### Neuropsychological measures administered to matched participants from Site 1 and Site 2

The California Verbal Learning Test, Children’s Version (CVLT-C), was administered to assess verbal learning and memory, which included the CVLT-C: List A Total (i.e., learning trials 1–5), short delay free recall, long delay free recall, and the recognition trial (i.e., recognition hits) [28]. The Purdue Pegboard Test ‘Both Hands’ score was used to measure fine motor dexterity [29]. The Stanford-Binet Intelligence Scales, Fourth Edition Vocabulary (verbal reasoning), Pattern Analysis (abstract/visual reasoning), Quantitative Reasoning (index measuring the ability to use quantitative reasoning to solve mathematical problems), Bead Memory (short-term memory), and Overall Intelligence Quotient (IQ) scales were used to assess general cognitive abilities [30]. The Beery-Buktenica Developmental Test of Visual-Motor Integration, Third Edition (VMI) was administered to assess visuomotor integration abilities [31]. The Woodcock-Johnson-Revised Tests of Achievement (WJ-R) Letter-Word Identification (single-word reading) and Calculation subtests (math calculation problem-solving) were used to assess academic achievement [32].

### Data analyses

#### Descriptive statistics and data preparation

Prior to conducting hypothesis tests, descriptive statistics were calculated to determine psychometric properties for all study variables. This included computing means and standard deviations. Variables were examined for outliers, defined as data points with *z*-score > 3.00 that did not belong to the normal distribution [33, 34]. Subsequently, the skewness of the variables was assessed. Variables demonstrating a skewness value greater than 2.1 were considered substantially deviated from normality. In such cases, appropriate transformations were applied to achieve approximately normal distributions.

#### Comparative analyses

To compare outcomes between sites, independent samples *t*-tests were completed. Given the relatively small number of comparison tests conducted, no adjustments were made to control for type 1 errors.

#### Power analysis

G*Power 3.1 was utilized to perform power analyses for our sample sizes. The analysis revealed ample statistical power to detect moderate to large effects. Power = 0.77 for detecting an effect size of *d* = 0.60.

#### Effect size interpretation

To aid in the interpretation of results, effect sizes are reported using Cohen’s *d*. The conventional interpretations are as follows: *d* = 0.20: Small effect; *d* = 0.50: Medium effect; *d* = 0.80: Large effect. This comprehensive approach to data analysis ensures robust and interpretable results, accounting for the statistical properties of our data and the power of our analyses.

## Results

### Preliminary analyses

Outliers were identified across three variables (Purdue Pegboard, SB Quantitative Reasoning, and WJ Letter-Word Identification). These outliers were corrected by changing the score to one unit greater than the next highest value, as recommended by Cohen and colleagues [35]. No variables were skewed based on predefined criteria (see the “Data analyses” section). Group comparisons for cognitive, motor, and academic outcomes are presented in Table 2.

**Table 2:** Site differences in cognitive, academic, and motor outcomes

Construct	Measure	Source	Site 1 (*N* = 41)	Site 2 (*N* = 41)	Cohen’s *d*
Learning and memory	CVLT-C List A Total^a^	Test data	38.94 (14.52)	35.04 (11.02)	0.50
	CVLT-C Short Delay^b^	Test data	− 0.94 (1.28)	− 1.34 (1.14)	0.33
	CVLT-C Long Delay^b^	Test data	− 0.97 (1.30)	− 1.27 (1.24)	0.24
	CVLT-C Recognition^b^	Test data	− 0.27 (1.01)	0.04 (0.91)	0.32
Fine motor dexterity	Purdue Pegboard^c^	Test data	**65.45 (25.91)**	**49.65 (33.56)**	**0.53**
General cognitive abilities	SB Vocabulary^a^	Test data	47.63 (9.09)	44.68 (8.56)	0.33
	SB Pattern Analysis^a^	Test data	42.80 (7.76)	41.17 (8.21)	0.20
	SB Quantitative Reasoning^a^	Test data	**45.53 (7.40)**	**41.95 (6.82)**	**0.50**
	SB Bead Memory^a^	Test data	44.75 (10.06)	40.63 (8.89)	0.43
	SB Overall IQ^c^	Test data	88.76 (15.64)	82.43 (14.22)	0.42
Visual-motor integration	VMI^c^	Test data	**80.20 (15.74)**	**73.21 (12.56)**	**0.49**
Academic abilities	WJ-R Letter-Word Identification^c^	Test data	**104.23 (20.36)**	**93.33 (22.33)**	**0.51**
	WJ-R Calculation^c^	Test data	**91.44 (23.93)**	**77.42 (23.83)**	**0.59**

Mean (*SD*) presented for continuous outcome data

Significant group differences (*p* <.05) are bolded

*CVLT-C *California Verbal Learning Test-Children’s Version, *SB * Stanford-Binet Intelligence Scales, Fourth Edition, *IQ * Intelligence Quotient, *VMI *The Beery-Buktenica Developmental Test of Visual-Motor Integration, Third Edition, *WJ-R *Woodcock-Johnson-Revised Tests of Achievement

Superscripts denote ^a^*T*-score ^b^*z*-score ^c^standard score

### Comparisons between Site 1 and Site 2

No significant differences were found between Site 1 and Site 2 across age, sex, race/ethnicity, or lesion level. However, a significant difference in shunt status was observed, with Site 1 having significantly fewer shunted patients (51%) compared to Site 2 (83%), *X*^2^ (1, 82) = 9.33, *p* = 0.002 (see Table 1).

Participants from Site 1 demonstrated significantly higher scores (i.e., better performance) than Site 2 on several measures: Purdue Pegboard, *t*(60) = 2.08, *p* = 0.042, Stanford-Binet Quantitative Reasoning, *t*(76) = 2.22, *p* = 0.030, VMI, *t*(76) = 2.16, *p* = 0.034, WJ Letter-Word Identification, *t*(77) = 2.27, *p* = 0.026, and WJ Calculation subtests, *t*(61) = 2.33, *p* = 0.012, than children in Site 2 (Table 2). These differences showed medium effect sizes. Although not statistically significant, effect sizes between 0.40 and 0.50 were also observed for CVLT-C List A Total, Stanford-Binet Bead Memory, and Stanford-Binet Overall IQ, with Site 1 scoring higher than Site 2.

## Discussion

This study provides valuable insights into the neurocognitive outcomes of patients with MMC across institutions with varying shunt placement rates. The findings challenge some previous assumptions about the relationship between shunting and cognitive outcomes in this population.

### Shunt placement and cognitive outcomes

MMC patients at Site 1 were shunted based on criteria first described in 2008 by Chakraborty et al. [5]. This approach resulted in a shunt rate of approximately 55%, significantly lower than the traditional rates of 72–96%. This more conservative approach to shunting aligns with recent trends, as an institution in Toronto reported a decrease in shunt rate from 77% in 1980–1989 to 63% in 1990–1999 [5, 36].

No guidelines currently exist for the treatment of asymptomatic ventriculomegaly in MMC patients. In 2019, the Congress of Neurological Surgeons published guidelines on persistent ventriculomegaly in MMC patients, citing insufficient evidence on the association between ventriculomegaly and decreased neurocognitive outcomes [21]. In premature intraventricular hemorrhage hydrocephalus patients, larger ventricles have been associated with decreased functional outcomes [37, 38]. However, this is compounded by severe prematurity and other birth complications [37, 38]. In MMC patients, larger ventricles have been correlated with decreased verbal and nonverbal capabilities [18]. However, studies in Ugandan and North American hydrocephalus patients with MMC have contradicted earlier findings [17, 19, 23]. In 2009, Warf et al. demonstrated similar cognitive findings between spina bifida patients treated with VP shunt and those treated with ETV/CPC, despite the latter having larger ventricles [22]. A recent prospective trial from HCRN reported minimal cognitive difference between MMC patients treated with VP shunt compared to ETV [23]. Additionally, a secondary analysis conducted on the MOMS cohort demonstrated better cognitive outcomes for patients who did not undergo CSF diversion, compared to those who did (for both postnatal and prenatally repaired groups) [39]. However, these studies compare outcomes following CSF diversion in compensated patients, which is not always reflective of the initial severity of hydrocephalus. In the MOMS follow-up study, patients who did not undergo CSF diversion had lower rates of callosal hypogenesis, lower lesion levels, and other indicators of less severe disease [39]. Further studies are needed to evaluate the impact of the initial severity of hydrocephalus on cognitive outcomes and define criteria for CSF diversion in this population.

### Cognitive performance across domains

**Fig. 3:**
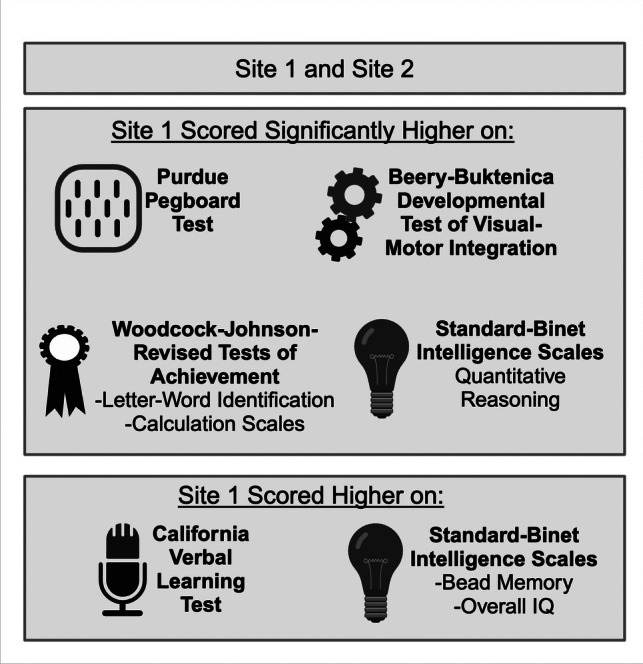
Differences in neurocognitive measures between Site 1 and Site 2. Findings included in the box where Site 1 “scored higher” were not statistically significant but represented medium effect sizes. Created in BioRender. Bowman, R. (2024) https://BioRender.com/i51o101

Our study demonstrates that patients from an institution with a lower shunt rate (Site 1) had comparable or even superior cognitive outcomes compared to those from higher shunt rate institutions (Site 2). Patients from Site 1 demonstrated significantly higher scores on several neurocognitive measures (Fig. 3): fine motor dexterity (Purdue Pegboard), cognitive abilities (Stanford-Binet Quantitative Reasoning), visual-motor integration (VMI), single-word reading (WJ-R Letter-Word Identification), and math calculation problem solving (WJ-R Calculation). Additionally, while not statistically significant, Site 1 patients showed trends (i.e., medium effect sizes) toward better performance on measures of higher verbal learning and memory. Our results suggest that a more conservative approach to shunt placement may not negatively impact cognitive outcomes and may even be associated with better performance in some domains.

Furthermore, several studies have demonstrated decreased longevity and cognitive functioning in MMC patients who underwent CSF diversion [40–42]. This may be reflective of several factors. First, shunt failure is common, and revision is associated with increased mortality and decreased long-term neurological outcomes [43, 44]. Additionally, patients who do not undergo CSF diversion treatment may represent a less severe disease presentation. These findings may represent an overtreatment of asymptomatic ventriculomegaly in MMC patients and the need for improved guidelines for neurosurgical intervention.

While our findings suggest potential benefits for a more conservative shunting approach, it is important to note that long-term neurocognitive impact in adulthood is unknown [5]. Further investigation is needed to establish evidence-based guidelines surrounding the treatment of hydrocephalus in MMC patients, specifically investigating treatment for asymptomatic ventriculomegaly.

## Limitations

While this study provides important information on outcomes across sites with different shunting rates, it also has several limitations. The retrospective nature of the study limits causal inferences. The sample size, while substantial for a relatively uncommon pediatric condition, may not capture the full spectrum of MMC presentations. This study was conducted using a historical database, and some data was not available, including head circumference, FOHR, or shunt type. Therefore, shunt rate at each institution was used as a proxy for the rate of ventriculomegaly as the exact rate of asymptomatic ventriculomegaly at each institution cannot be determined. Moreover, we do not have data on the extent of ventricular size or severity of the Chiari II malformation. This study aims to compare management style between two institutions. However, this study provides important data that can lay the groundwork for further discussion and future studies on CSF diversion management for MMC patients.

## Conclusion

We conducted a matched cohort study comparing neuropsychological outcomes in MMC patients between institutions with different shunt rates. This study challenges the historical approach toward shunt placement in youth with MMC and suggests that conservative approaches to CSF diversion may lead to comparable or even improved cognitive outcomes. However, future studies are needed to directly compare outcomes of patients with MMC and asymptomatic ventriculomegaly.

## Data Availability

Data can be accessed upon request by contacting the corresponding author.
